# Crosstalk between advanced glycation end products (AGEs)-receptor RAGE axis and dipeptidyl peptidase-4-incretin system in diabetic vascular complications

**DOI:** 10.1186/s12933-015-0176-5

**Published:** 2015-01-13

**Authors:** Sho-ichi Yamagishi, Kei Fukami, Takanori Matsui

**Affiliations:** Department of Pathophysiology and Therapeutics of Diabetic Vascular Complications, Kurume University School of Medicine, 67 Asahi-machi, Kurume, 830-0011 Japan; Department of Medicine, Kurume University School of Medicine, Kurume, 830-0011 Japan

**Keywords:** AGEs, RAGE, Incretin, DPP-4, Oxidative stress

## Abstract

Advanced glycation end products (AGEs) consist of heterogenous group of macroprotein derivatives, which are formed by non-enzymatic reaction between reducing sugars and amino groups of proteins, lipids and nucleic acids, and whose process has progressed at an accelerated rate under diabetes. Non-enzymatic glycation and cross-linking of protein alter its structural integrity and function, contributing to the aging of macromolecules. Furthermore, engagement of receptor for AGEs (RAGE) with AGEs elicits oxidative stress generation and subsequently evokes proliferative, inflammatory, and fibrotic reactions in a variety of cells. Indeed, accumulating evidence has suggested the active involvement of accumulation of AGEs in diabetes-associated disorders such as diabetic microangiopathy, atherosclerotic cardiovascular diseases, Alzheimer’s disease and osteoporosis. Glucagon-like peptide-1 (GLP-1) and glucose-dependent insulinotropic polypeptide (GIP) are incretins, gut hormones secreted from the intestine in response to food intake, both of which augment glucose-induced insulin release, suppress glucagon secretion, and slow gastric emptying. Since GLP-1 and GIP are rapidly degraded and inactivated by dipeptidyl peptidase-4 (DPP-4), inhibition of DPP-4 and/or DPP-4-resistant GLP-1 analogues have been proposed as a potential target for the treatment of diabetes. Recently, DPP-4 has been shown to cleave multiple peptides, and blockade of DPP-4 could exert diverse biological actions in GLP-1- or GIP-independent manner. This article summarizes the crosstalk between AGEs-RAGE axis and DPP-4-incretin system in the development and progression of diabetes-associated disorders and its therapeutic intervention, especially focusing on diabetic vascular complications.

## Introduction

According to the recent report of Diabetes Atlas, about 380 million people have diabetes, and the number is still increasing in almost every country, especially in Asian areas [1]. Diabetic micro- and macroangiopathy are devastating vascular complications of diabetes. From the standpoint of patients’ quality of life, diabetes is in a sense one of the cardiovascular diseases. Indeed, diabetic nephropathy and retinopathy are one of the leading causes of end-stage renal failure and acquired blindness, respectively, which in concert with neuropathy could account for disabilities in patients with diabetes [1-3]. Atherosclerotic cardiovascular diseases (CVD) account for about 60% of death in diabetic subjects [4]. Furthermore, even after adjusting several risk factors, including systolic blood pressure, lipid levels, C-reactive protein and fibrinogen values, socioeconomic status, or estimated glomerular filtration rate, hazard ratios among diabetic patients as compared with non-diabetic people were 1.8 for death from any cause, 2.3 from CVD, 1.7 from non-CVD, and 1.25 from several cancers [4]. On the other hand, the association of diabetes with mortality was considerably reduced after adjusting for fasting glucose or glycated hemoglobin levels. At fasting glucose levels above 100 mg/dl, hazard ratio of vascular, non-vascular and cancer deaths for higher glucose levels, assessed in increments of 18 mg/dl, were 1.13, 1.10 and 1.05, respectively [4]. Along with this, life expectancy is reduced by more than 20 years in middle-aged people of type 1 diabetes and by up to 10 years in middle aged-type 2 diabetic patients compared with non-diabetic subjects [4-7]. 40-years old diabetic subjects without known CVD at the time of enrollment died about 6.3 years younger than non-diabetic subjects [4]. As a result, average life span of diabetic patients is about 10-15-years shorter than that of non-diabetic subjects. Moreover, the increased risk for Alzheimer’s disease and osteoporosis is also observed in diabetic patients [8,9]. These observations indicate that the risk of aging-related disorders and premature death is high in diabetic subjects and that cumulative hyperglycemic exposure might be directly involved in the high mortality rate in patients with diabetes. Since accumulation of advanced glycation end products (AGEs) could reflect cumulative historical diabetic exposure, and thereby, play a role in various diabetes and/or aging-associated disorders [2,8,9], blockade of harmful effects of AGEs might be a novel therapeutic target for organ protection in diabetes. Further, inhibition of dipeptidyl peptidase-4 (DPP-4), a responsible enzyme that mainly degrades incretins such as glucagon-like peptide-1 (GLP-1) and glucose-dependent insulinotropic polypeptide (GIP), has been shown not only to improve glycemic control, but also to exert diverse beneficial actions in GLP-1- or GIP-independent manner [10]. So, this article summarizes the crosstalk between AGEs-receptor for AGEs (RAGE) axis and DPP-4-incretin system in the development and progression of diabetes-associated disorders and its therapeutic intervention, especially focusing on diabetic vascular complications. In the present review, literature searches were undertaken in Medline by the PubMed interface. Non-English language articles were excluded. Key words ((glycation or RAGE) and (DPP-4 or GLP-1 or GIP or incretins) have been used to select the articles.

### Metabolic memory, legacy effect and hyperglycemic curse

The Diabetes Control and Complications Trial-Epidemiology of Diabetes Interventions and Complications (DCCT-EDIC) Research, has revealed that the reduction in the risk of progressive retinopathy and nephropathy resulting from intensive therapy in patients with type 1 diabetes persists for at least several years after the DCCT trial, despite increasing hyperglycemia [11,12]. Recently, intensive diabetes treatment has been shown to yield durable retinal and renal benefits that persist for at least 18 years after its application [13,14], and the prevalence and incidence of diabetic peripheral neuropathy and cardiovascular autonomic neuropathy have remained significantly lower in the DCCT intensive therapy group compared with the DCCT conventional therapy group through EDIC year 13-14 [15]. In addition, intensive therapy during the DCCT resulted in decreased progression of intima-media thickness (IMT) and subsequently reduced the risk of nonfatal myocardial infarction, stroke, or death from cardiovascular disease by 57% 11 years after the end of the trials [16,17]. The major adverse effect of intensive therapy was a threefold increased risk of hypoglycemia, which was not associated with a decline in cognitive function or quality of life [18]. Compared with intensive therapy group subjects who remained weight stable during DCCT, excess gainers maintained greater body mass index and waist circumference, had greater IMT of carotid artery, and trended toward greater coronary artery calcium scores [19]. Increasing frequency of a family history of diabetes mellitus, hypertension, and hyperlipidemia was associated with greater IMT in patients of intensive but not conventional treatment group [19]. These observations suggest that genetic susceptibility to weight gain during DCCT might be a marker of worsening atherosclerosis in the intensive therapy-received subjects during follow-up in EDIC.

Furthermore, a follow-up study of United Kingdom Prospective Diabetes Study (UKPDS), called UKPDS80, has also shown that benefits of an intensive therapy in patients with type 2 diabetes are sustained after the cessation of the trial [20]. In this study, despite an early loss of glycemic differences between intensive and conventional therapy, a continued reduction in microvascular risk and emergent risk reductions for myocardial infarction and death from any cause were observed during 10 years of post-trial follow-up [20]. These findings demonstrate that so-called ‘metabolic memory’, in other words, ‘hyperglycemic curse’ may cause chronic abnormalities in diabetic vessels that are not easily reversed, even by subsequent, relatively good control of blood glucose, thus suggesting a long-term beneficial influence of early metabolic control, *that is*, legacy effect, on the risk of diabetic retinopathy, nephropathy, CVD and death in both type 1 and type 2 diabetic patients. Chronic persistent hyperglycemia is a major initiator of vascular complications of diabetes [2,3]. Various hyperglycemia-elicited metabolic and hemodynamic derangements such as increased formation of AGEs, enhanced production of reactive oxygen species (ROS) and activation of the renin-angiotensin system (RAS) and protein kinase C have been proposed to contribute to the characteristic histopathological changes within the diabetic vessels [2,3,21,22]. The metabolic memory is linked not only to AGEs-RAGE axis but also to some other pathways [23-26]. Indeed, activation of p66shc adaptor protein is part of a signal transduction pathway relevant to hyperglycemia vascular damage [23], and p66shc oxidizes cytochrome C and generates ROS in response to stress signals through a protein kinase C-dependent pathway [24]. Moreover, recent finding has highlighted that immunization with AGEs inhibits atherosclerosis progression in diabetic apoE and LDLR null mice [25], thus suggesting the involvement of specific humoral and cell-mediated immune responses in diabetic macroangiopathy. Further, AGEs have also been shown to induce cardiomyocyte autophagy by, at least in part, inhibiting the phosphatidylinositol 3-phosphate kinase/Akt/mammalian target of rapamycin pathway via RAGE [26]. Since these pathways are interrelated with each other, and therefore, it remains unclear which molecular pathway is the most crucial one for causing vascular damage in diabetes. However, the biochemical nature and mode of action of AGEs are most compatible with the concept of ‘metabolic memory’ [27-31].

### Activation of the AGEs-RAGE axis and metabolic memory

AGEs are formed by the Maillard process, a non-enzymatic reaction between reducing sugars and the amino groups of proteins, lipids and nucleic acids that contributes to the aging of macromolecules [27-31]. Under hyperglycemic and/or oxidative stress conditions, this process begins with the conversion of reversible Schiff base adducts to more stable, covalently-bound Amadori rearrangement products [27-31]. Over the course of days to weeks, these Amadori products undergo further rearrangement reactions to form the irreversibly-crosslinked moieties termed AGEs. About 10% of Amadori products could move to the irreversible process [32]. The formation and accumulation of AGEs have been known to progress in a normal aging process and at an accelerated rate under diabetes [27-31]. In addition, AGEs are slowly degraded and remain for a long time in diabetic vessels even after glycemic control has been improved [33,34]. Indeed, tissue accumulation levels of pentosidine have persisted for long periods of time in patients after kidney or kidney-pancreas transplantation, despite consistent decreases in plasma levels of pentosidine [34]. These observations indicate that formation and accumulation of AGEs may continue in diabetic and/or renal failure patients after successful treatment of blood glucose, kidney or kidney-pancreas transplantation.

Non-enzymatic glycation and cross-linking of proteins such as collagen impair its structural integrity and function [27-31]. In addition, numerous studies have reported that AGEs and RAGE interaction elicit oxidative stress generation in various types of cells and subsequently evoke proliferative, inflammatory, thrombogenic and fibrotic reactions, thereby playing an important role in the development and progression of diabetes-associated disorders such as diabetic microangiopathy, atherosclerotic cardiovascular diseases, Alzheimer’s disease and osteoporosis [8,9,22,35-40]. Moreover, AGEs are reported to up-regulate RAGE expression and induce sustained activation of nuclear factor-κB (NF-κB) [41-43]. Therefore, it is conceivable that the AGEs-RAGE-induced oxidative stress generation further potentiates the formation and accumulation of AGEs and subsequent RAGE overexpression [41-48]. So, besides the irreversible nature of AGEs, the positive feedback loops between AGEs and RAGE-downstream pathways could make a vicious cycle, thus providing a mechanistic basis for understanding why there could exist the phenomenon of ‘metabolic memory’ in vascular complications in diabetes.

Diet or smoking is a major environmental source of AGEs in humans [49-53]. About 10% of exogenously derived AGEs were absorbed, two-thirds of which remained in the body [49-53]. So, overintake of food-derived AGEs or smoking could increase circulating and tissue accumulation levels of AGEs in non-diabetic individuals [49-53]. Former smokers remain at an increased risk for developing lung cancer and CVD even years after they stop smoking [53]. The possible reason why it had the interesting analogy to the phenomenon of metabolic memory is that smoking-derived AGEs may have carry-over effects on the development and progression of lung cancer [53]. Recent epidemiological studies have shown that the incidence of cancers, including lung cancer is increased in diabetic patients, especially poorly controlled diabetes [4]. Furthermore, we have very recently found that even after adjusting for potential confounding risk factors, serum levels of AGEs could predict the risk of rectal cancer in an European Prospective Investigation into Cancer and Nutrition Cohort (unpublished data). Therefore, accumulation of endogenously and/or exogenously derived AGEs might partly explain the increased risk of various cancers in diabetes.

Food-derived AGEs have been shown to promote the aging-associated tissue damage as well [49-52]. Nutrient composition, temperature and method of cooking can affect the formation of AGEs in foods; fats or meat-derived products processed by high heat such as broiling, frying and oven-roasting contain more AGEs than carbohydrates boiled for longer periods [49-52]. Moreover, isocaloric restriction of dietary AGEs has been shown to decrease circulating AGEs levels and inflammatory biomarkers, improve endothelial dysfunction and restore the decreased levels of sirtuin 1, an anti-aging molecule in diabetic patients [54,55]. In addition, AGEs-, but not calorie-restricted foods can expand lifespan in mice, while calorie-, but not AGEs-restricted diets can not [56]. These observations suggest that restriction of food-derived AGEs rather than calorie itself might be a therapeutic target for anti-aging medicine. Taken together, blockade of the AGEs-RAGE axis might be a novel therapeutic target for slowing down the aging process and preventing diabetes-associated disorders.

### Incretins and DPP-4

Incretins such as GLP-1 and GIP are gut hormones secreted from L and K cells in the intestine in response to food intake, respectively [57,58]. Since GLP-1 and GIP augment glucose-induced insulin release from pancreatic β-cells, suppresses glucagon secretion, and slows gastric emptying [57,58], incretins have been proposed as a potential therapeutic target for the treatment of patients with diabetes. However, native GLP-1 and GIP have a very short half-life because they are rapidly degraded and inactivated by proteolytic enzyme, DPP-4 [59,60], and GIP is also recognized as an obesity-promoting factor in rats fed a high-fat diet [61]. Therefore, at present, DPP-4 inhibitors and/or DPP-4-resistant GLP-1 analogues rather than native GLP-1 are clinically used as a GLP-1-based medicine for the treatment of diabetic subjects.

The biological actions of GLP-1 on pancreatic cells are mainly mediated by high-affinity GLP-1 receptor (GLP-1R) [57]. In addition, GLP-1R is shown to exist in extra-pancreatic tissues, including brain, peripheral nervous system, kidney, heart and vasculature [62,63]. Furthermore, recently, DPP-4 has been shown to cleave multiple peptides, and blockade of DPP-4 could exert diverse biological actions in a variety of cells and tissues [64,65]. These observations suggest that DPP-4 inhibitors could act on cardiovascular, renal-retinal and nervous systems, and bone in both GLP-1-dependent and GLP-1-independent manners. Therefore, in the following section, we review the crosstalk between AGEs-RAGE axis and DPP-4-incretin system in the development and progression of diabetes-associated disorders.

### Crosstalk between AGEs-RAGE axis and DPP-4-incretin system

**Figure 1:**
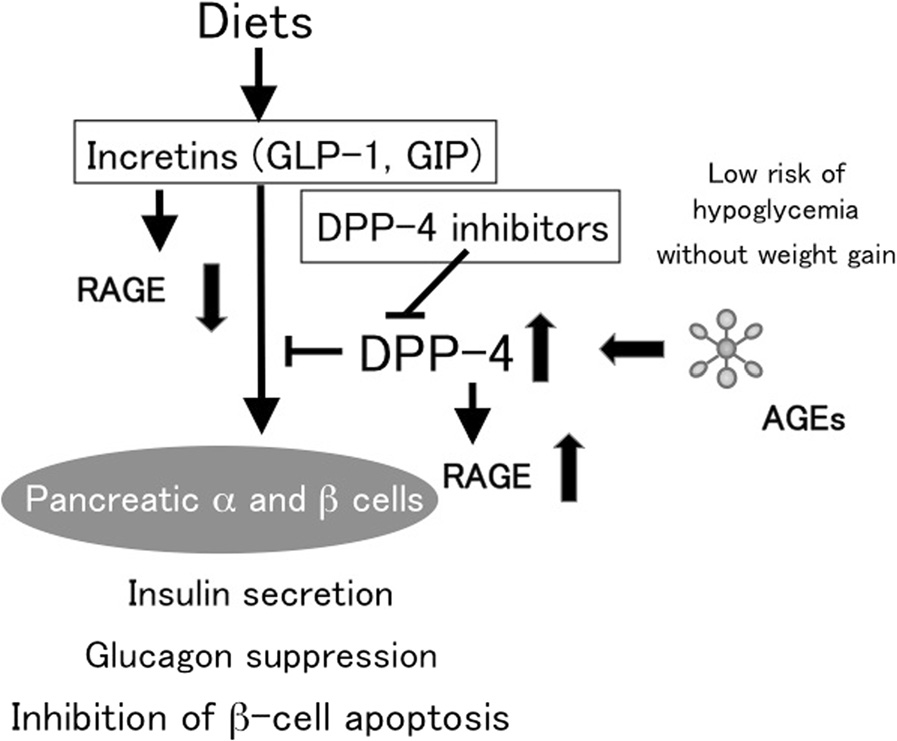
**Effects of incretins on pancreatic α and β cells.**

1. Atherosclerotic CVD
  Impaired endothelial cell (EC) growth and enhanced EC apoptosis are postulated to play a central role in the pathogenesis of early phase of atherosclerosis [66]. Atherogenic properties of AGEs could be partly attributed to its pro-apoptotic effects on ECs [67-69]. Furthermore, recent clinical investigations implicate the role of AGEs in impaired EC repair in atherosclerosis [70-73]. Diabetes is associated with endothelial dysfunction, decreased endothelial progenitor cells (EPCs) function and mobilization, which could contribute to accelerated atherosclerosis and increased risk for CVD in diabetic patients [70]. AGEs enhance apoptosis and suppress migration and tube formation of late EPCs through the interaction with RAGE via down-regulation of Akt and cycloxygenase-2 [71]. AGEs have also been shown to cause a reduction of length growth and EPC incorporation into the sprouts in association with RAGE overexpression and p38 mitogen-activated protein kinase (MAPK) activation [72]. Moreover, AGEs-modification of vascular substrates impair vascular repair by inhibiting EPC adhesion, spreading and migration via glycation of Arg-Gly-Asp motif of fibronectin [73]. In addition, we have found that serum levels of AGEs are inversely associated with number and migratory activity of EPCs in apparently healthy subjects [74]. Skin autofluorescence, an established non-invasive measure of tissue AGEs accumulation, but not serum levels of pentosidine, was independently associated with low circulating EPCs in subjects with end-stage renal failure [75]. Since GLP-1 was reported to protect against AGEs-induced apoptotic cell death of ECs in association with increased ratio of Bcl-2/Bax, reduced cytochrome C levels and suppressed caspase-3 and caspase-9 activity [76], GLP-1 might promote re-endothelialization following vascular injury, thereby slowing the development and progression of atherosclerosis. Indeed, vildagliptin significantly increased GLP-1 levels and reduced vascular senescence in Zucker diabetic fatty rats [77]. In addition, sitagliptin, an inhibitor of DPP-4 was reported to increase circulating EPCs in type 2 diabetic patients with concomitant up-regulation of stromal-derived factor-1α as well [78].
  There is a growing body of evidence, ranging from *in vitro*-experiments to pathologic analysis to epidemiologic studies, that atherosclerosis is intrinsically an inflammatory disease [79-82]. Pro-inflammatory cytokines such as tumor necrosis factor-α and interleukin-1 have been shown to cause endothelial dysfunction, an initial step of atherosclerosis [79-82]. Furthermore, atherosclerotic plaques contain numerous inflammatory cells, particularly macrophages, which could secrete a variety of growth factors, cytokines and enzymes and subsequently contribute to the weakening of the fibrous cap of the plaques, thereby leading to the development of acute coronary syndromes [82-85].
  Binding of AGEs to RAGE results in generation of intracellular ROS generation and subsequent activation of the redox-sensitive transcription factor, NF-κB in vascular wall cells, which could promote a variety of atherosclerosis/inflammation-related gene expression such as monocyte chemoattractant protein-1 (MCP-1), vascular cell adhesion molecule-1 (VCAM-1), intercellular adhesion molecule-1 (ICAM-1), and plasminogen activator inhibitor-1 (PAI-1) [41-49]. MCP-1 plays an important role in the early phase of atherosclerosis by initiating monocyte recruitment to the vessel wall, and its expression is elevated in human atherosclerotic plaques [86,87]. The selective targeting of CCR2, the receptor for MCP-1, markedly decreases atheromatous lesion formation in apolipoprotein E knockout mice [86,87]. One early phase of atherosclerosis involves the firm adhesion of inflammatory cells to ECs, whose process is mainly mediated by ICAM-1 and VCAM-1 [88-90]. Moreover, attenuated fibrinolytic activity due to increased PAI-1 levels is highly prevalent in diabetic patients, thus contributing to the increased risk of atherothrombosis and CVD in these subjects [91,92]. These observations further suggest the active participation of AGEs-RAGE axis in accelerated atherosclerosis in diabetes.
  We have found that GLP-1 blocks the AGE-induced up-regulation of VCAM-1 mRNA levels in human umbilical vein ECs (HUVECs) by suppressing RAGE expression and subsequent ROS generation [93]. Since GLP-1R was expressed in HUVECs and that small interfering RNAs (siRNAs) raised against GLP-1R inhibited the GLP-1-induced down-regulation of RAGE mRNA levels, our present study suggests that GLP-1 could directly act on ECs via GLP-1R and it might work as an anti-inflammatory agent against AGEs by reducing RAGE expression [93]. Furthermore, an analogue of cyclic AMP, 8-Br-cAMP mimicked the effects of GLP-1 on RAGE gene expression in HUVECs. We have previously shown that AGEs decrease the intracellular cyclic AMP levels in ECs and that cyclic AMP agonists such as dibutyryl cyclic AMP reduce the AGEs-RAGE-evoked PAI-1 production [94]. In addition, GIP receptor (GIPR) was also expression in HUVECs, and GIP, an analogue of cyclic AMP or an inhibitor of NADPH oxidase inhibited the AGEs-induced ROS generation in HUVECs [94]. GIP reduced gene and protein expression of RAGE and subsequently decreased VCAM-1 and PAI-1 mRNA levels in AGEs-exposed HUVECs. An anti-oxidant *N*-acetylcysteine mimicked the effects of GIP on HUVECs. Taken together, these findings suggest the involvement of cyclic AMP in the AGEs signaling pathways and that AGEs-RAGE-mediated NADPH oxidase is a molecular target for anti-inflammatory effects of GLP-1-GLP-1R- or GIP-GIPR-cyclic AMP axis in AGEs-exposed HUVECs. Vildagliptin treatment inhibited the increase in AGEs, RAGE and oxidative stress marker, 8-hydroxydeoxyguanosine levels of thoracic aorta of obese and type 2 diabetic rats, which were in association with decreased gene expression of MCP-1, VCAM-1 and PAI-1 and suppressed activity of NF-κB [95]. Further, vildagliptin reduced oxidative stress generation and decreased inflammatory, fibrotic and thrombogenic gene expression (ICAM-1, transforming growth factor- β (TGF-β), and PAI-1) in thoracic aorta of streptozotocin-induced diabetic rats, although it did not affect blood glucose levels in type 1 diabetic animals [96]. These findings suggest the glucose-lowering independent beneficial effects of DPP-4 inhibitors on experimental diabetic macroangiopathy.
  AGEs formed on the extracellular matrix results in decreased elasticity of vasculatures, and quench nitric oxide (NO), which could mediate defective endothelium-dependent vasodilatation, a surrogate marker of CVD in diabetes [97,98]. AGEs not only decreased endothelial NO synthase (eNOS) mRNA levels in ECs, but also reduced NO bioavailability by inactivating NO to form peroxynitrite via ROS generation [99]. We have previously shown that GLP-1 modestly restores decreased mRNA levels of eNOS in AGEs-exposed HUVECs, whose effect was significantly potentiated by the simultaneous treatment with sitagliptin [100]. GLP-1 infusion has also been shown to improve endothelial-dependent vasodilation in patients with type 2 diabetes, but not healthy subjects [101].
  DPP-4 dose-dependently increased ROS generation and RAGE gene expression in HUVECs, which were prevented by an inhibitor of DPP-4, linagliptin [47]. Mannose 6-phosphate (M6P) or neutralizing antibody raised against M6P/insulin-like growth factor II receptor (M6P/IGF-IIR) completely blocked the ROS generation in DPP-4-exposed HUVECs, whereas surface plasmon resonance revealed that DPP-4 bound to M6P/IGF-IIR at the dissociation constant of 3.59 × 10^−5^ M [47]. AGEs or hydrogen peroxide increased soluble DPP-4 production released from HUVECs, which was prevented by *N*-acetylcysteine, neutralizing antibody directed against RAGE or linagliptin [47]. Linagliptin significantly inhibited the AGE-induced ROS generation, RAGE, ICAM-1 and PAI-1 gene expression in HUVECs [47]. These findings suggest that the AGEs-RAGE-induced ROS generation stimulates the release of DPP-4 from ECs, which could in turn act on ECs directly via the interaction with M6P/IGF-IIR, further potentiating the deleterious effects of AGEs. The blockade by linagliptin of positive feedback loop between AGE-RAGE axis and DPP-4 might be a novel therapeutic target for vascular injury in diabetes (Figures 1 and 2).

**Figure 2:**
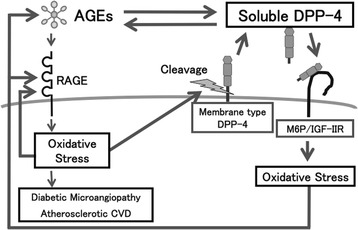
**Crosstalk between the AGEs-RAGE axis and DPP-4.**
  We have recently found that serum DPP-4 levels are independently correlated with HbA1c and AGEs in 432 outpatients who did not receive DPP-4 inhibitors [102]. Furthermore, AGEs enhanced the expression of DPP-4 in, and its release from cultured proximal tubular cells, a major cell type that expressed DPP-4 [102,103]. Since serum AGEs levels are positively correlated with soluble forms of RAGE and VCAM-1 (sRAGE and sVCAM-1) in diabetic patients [104-106], AGEs might promote the proteolytic cleavage of plasma membrane-bound proteins such as RAGE, VCAM-1 and DPP-4. Given the facts that serum DPP-4 activity is largely associated with circulating DPP-4 levels [59,107] and that 20% of incretins derived from gastrointestinal tract are still alive in the blood pool [108,109], AGEs may impair the effects of incretins via induction of DPP-4, further augmenting the formation and accumulation of AGEs, making a vicious cycle in poorly controlled diabetes (Figures 1 and 2). The relatively weak clinical benefit of vildagliptin in patients with a long history of diabetes could be partly ascribed to DPP-4 induction evoked by AGEs [110]. In other words, serum AGEs levels may be a clinical biomarker to identify which type 2 diabetic patients will respond less to the treatment with DPP-4 inhibitors.
2. Diabetic cardiomyopathy
  Diabetic cardiomyopathy is characterized by early impairments in cardiac diastolic dysfunction, accompanied by the development of cardiomyocyte hypertrophy and myocardial stiffness, which are independent of coronary artery disease and hypertension [105-107]. AGEs-RAGE axis plays a role in the pathogenesis of diabetic cardiomyopathy via inducing endothelial dysfunction, altering calcium handing/contractility, and evoking inflammatory, fibrotic and pro-apoptotic reactions in the myocardium [111-113]. Myocardium of 10-week old db/db^−/−^ mice, a model of diabetes and obesity, exhibited a marked increase of phosphorylation of AMP-activated protein kinase and acetyl-CoA carboxylase as well as membrane expression of fatty acid translocase, thus suggesting the altered fatty acid metabolism in cardiomyocytes of these animals [114]. Furthermore, myocardial fibrosis and increased expression levels of TGF − β and AGEs were observed in db/db^−/−^ mice [114]. Sitagliptin improved glucose tolerance and reduced weight gain in db/db^−/−^ mice, which were associated with amelioration of these pathological changes in cardiomyocytes [114]. Exendin-4, a GLP-1R agonist significantly inhibited hyperglycemia-induced RAGE expression and apoptosis of cardiomyocytes, and improved cell viability as well [115].
  Hypertension also induces cardiac dysfunction, calcium dysregulation, and arrhythmogenesis [116]. Sitagliptin imporved cardiac electrophysiological characteristics and calcium dysregulation in spontaneous hypertensive rats by reducing RAGE and angiotensin II type 1 receptor levels via its anti-inflammatory properties [116].

3) Diabetic nephropathy
  Diabetic nephropathy is characterized by functional and structural changes in the glomerulus such as glomerular hyperfiltration, thickening of glomerular basement membrane, and an expansion of extracellular matrix in the mesangial areas [117,118]. It ultimately progresses glomerular sclerosis associated with an increased urinary excretion rate of albumin and renal dysfunction [117,118]. In addition, although characteristic histological changes of diabetic nephropathy were diffuse and nodular glomerulosclerosis [119], it has been also recognized that changes within tubulointerstitium are more important than glomerulopathy in terms of renal dysfunction in diabetic nephropathy [120,121].
  Engagement of RAGE with the ligand AGEs elicits oxidative stress generation and resultantly evokes inflammatory and fibrotic reactions in the kidney cells, thereby causing progressive alteration in renal architecture and loss of renal function associated with tubular injury in diabetes [117,122]. Furthermore, diabetic homozygous RAGE null mice failed to develop significantly increased mesangial matrix expansion or thickening of the glomerular basement membrane [123]. Deletion of RAGE is also reported to prevent diabetic nephropathy in the OVE26 type 1 mouse, a model of progressive glomerulosclerosis and decline of renal function [124].
  AGEs induce apoptotic cell death and stimulate expression of vascular endothelial growth factor (VEGF) in human cultured mesangial cells, a counterpart of pericytes in the kidney [125]. Mesangial cells occupy a central anatomical position in the glomerulus, playing crucial roles in maintaining structure and function of glomerular capillary tufts [120]. They actually provide structural support for capillary loops and modulate glomerular filtration by its smooth muscle activity [126-128]. Therefore, the AGEs-induced mesangial apoptosis and dysfunction may contribute in part to glomerular hyperfiltration, an early renal dysfunction in diabetes. Antibody directed against VEGF has been shown to improve hyperfiltration and albuminuria in experimental diabetic nephropathy, and urinary VEGF level is correlated with albuminuria in type 2 diabetic patients, thus suggesting the pathological role of VEGF in hyperpermeability and albuminuria in diabetic nephropathy [129].
  AGEs stimulate MCP-1 expression in mesangial cells as well [124]. Increased MCP-1 expression associated with monocyte infiltration in mesangium has been observed in the early phase of diabetic nephropathy [130]. Plasma MCP-1 level was associated with albumin excretion rate in patients with type 1 diabetes, a marker of early diabetic nephropathy [131]. Further, urinary MCP-1/creatinine ratios in type 2 diabetic patients with microalbuminuria were much higher than those in normal controls, and intensive insulin treatment significantly decreased the urinary MCP-1/creatinine ratios [132]. Moreover, selective targeting of MCP-1, was shown to markedly decrease albuminuria, renal injury and fibrosis in streptozotocin-induced diabetic nephropathy [133]. These observations suggest that AGEs accumulation in the glomeruli could be implicated in inflammatory and fibrogenic reactions in diabetic nephropathy as well via promoting the secretion of MCP-1 by mesangial cells.
  GLP-1R is expressed in mesangial cells and proximal tubular cells [103,134,135]. GLP-1 suppresses RAGE gene and protein expression and subsequently inhibits the AGEs-induced ROS generation and MCP-1 expression in human cultured mesangial cells [136]. siRNAs directed against GLP-1R reduced GLP-1R levels and inhibited the suppressive effects of GLP-1 on RAGE mRNA levels in mesangial cells [136]. In addition, as the case in HUVECs, an analogue of cAMP, 8-bromo-cAMP mimicked the effects of GLP-1 on RAGE gene expression, ROS generation and MCP-1 mRNA levels in mesangial cells. These findings suggest that GLP-1 could inhibit the harmful effects of AGEs-RAGE axis on mesangial cells via GLP-1R-mediated cAMP elevation.
  NO is a multifunctional molecule critical to a number of physiological and pathological processes in humans [136,137]. NO not only inhibits inflammatory-proliferative reactions in vascular wall cells and kidney, but also exerts anti-thrombogenic and EC protective properties *in vivo* [136,137]. Therefore, impaired production and/or bioavailability of NO are considered to play a role in vascular complications in diabetes such as diabetic nephropathy and CVD [136-139]. Indeed, circulating level of asymmetric dimethylarginine (ADMA), an endogenous NO synthase inhibitor is increased in early diabetic nephropathy in type 1 diabetes and associated with future cardiovascular events in these subjects [140]. Furthermore, serum levels of AGEs were positively associated with sRAGE and ADMA in patients with chronic kidney disease [141]. Plasma ADMA levels were positively associated with serum AGEs level and inversely correlated with endothelial function determined by flow-mediated vasodilatation [142], thus suggesting the active involvement of AGEs-RAGE system in the elevated levels of ADMA in humans. We have recently found that GLP-1 inhibits the AGEs-induced RAGE gene expression, ROS generation and gene expression of protein arginine methyltransfetase-1 (PRMT-1), a rate-limiting enzyme for ADMA generation and subsequently reduces ADMA levels in cultured human proximal tubular cells, all of which were blocked by siRNAs raised against GLP-1R. [135]. In addition, neutralizing antibody raised against RAGE or *N*-acetylcysteine inhibited the AGEs-induced tubular cell gene expression of PRMT-1. Furthemore, continuous intraperitoneal infusion of GLP-1 analogue, exendin-4 inhibited renal RAGE gene expression, reduced urinary excretion level of 8-hydroxy-2’-deoxyguanosine (8-OHdG), an oxidative stress marker, decreased PRMT-1 mRNA levels and ADMA generation in the kidney of type 1 diabetic animals, in association with improvement of glomerular hypertrophy, macrophage infiltration into the glomeruli, and glomerular and tubulointerstitial fibrosis [135]. These observations suggest that RAGE gene suppression in tubular cells could be a central mechanism by which GLP-1 inhibited ADMA levels in the kidney of early phase of experimental diabetic nephropathy. AGEs decreased mRNA levels of dimethylarginine dimethylaminohydrolase-II, a responsible enzyme for ADMA degradation and suppressed its enzymatic activity, and resultantly increased ADMA levels in HUVECs, all of which were completely blocked by an anti-oxidant, *N*-acetylcysteine [142].
  There is a growing body of evidence that renin-angiotensin system plays a role in diabetic nephropathy [143,144]. We have found that GLP-1 blocked the angiotensin II-induced superoxide generation, NF-κB activation, up-regulation of mRNA levels of ICAM-1 and PAI-1 in mesangial cells, all of which were prevented by the treatments with H-89, an inhibitor of protein kinase A [145], thus suggesting that GLP-1 could block the angiotensin II-induced mesangial cell injury by inhibiting superoxide-mediated NF-κB activation via protein kinase A pathway [145].
  Chronic hyperglycaemia induces a significant increase in DPP-4 activity in type 1 and type 2 diabetes [146]. In accordance with the observation, we have found that serum levels of DPP-4 are significantly elevated in streptozotocin-induced diabetic rats compared with control rats [147]. Although linagliptin treatment for 2 weeks did not improve hyperglycemia in diabetic rats, linagliptin significantly reduced AGEs levels, RAGE gene expression and 8-OHdG in the kidney of diabetic rats [147]. Furthermore, linagliptin significantly reduced albuminuria, renal ICAM-1 mRNA levels and lymphocyte infiltration into the glomeruli of diabetic rats [147]. The findings have extended our previous observations showing that linagliptin blocked the crosstalk between DPP-4 and AGEs-RAGE axis in ECs [47]. Linagliptin could exert beneficial effects on diabetic nephropathy partly by blocking the AGEs-RAGE-evoked oxidative stress generation in the kidney of streptozotocin-induced diabetic rats. Furthermore, we have very recently found that linagliptin contains xanthine scaffold structure, which could inhibit xanthine oxidase activity *in vitro* and reduce uric acid levels in type 2 diabetic patients [148,149]. The anti-oxidative unique properties of this drug might also be involved in the blockade of vicious cycle between ROS generation and RAGE gene induction in diabetic nephropathy. Moreover, we have found that DPP-4 inhibitor alogliptin treatment blocks the AGEs-RAGE axis and resultantly reduces albuminuria in type 2 diabetes patients [150].
4) Diabetic retinopathy
  We have previously shown that vildagliptin treatment for 10 weeks prevented the increase in body weight and decreased average fasting blood glucose in OLETF rats, an animal model of type 2 diabetes with obesity [151]. Further, vildagliptin treatment was found to completely inhibit the increase in angiogenic, inflammatory and thrombogenic gene expression (VEGF, ICAM-1 and PAI-1) in the retinas of OLETF rats [151]. Exendin-4 and GLP-1 decreased RAGE levels in AGEs-exposed human retinal pigment epithelial cells and made these cells more resistant to harmful effects of AGEs, leading to suppression of ICAM-1 and VCAM-1 levels [152], thus suggesting the clinical utility of DPP-4 inhibitors and/or GLP-1-based medicine for the treatment of obese type 2 diabetes, including diabetic retinopathy.
  The effects of DPP-4 inhibition on microvascular complications were recently thoroughly described by Avogaro [153]. Experimental findings and preliminary clinical data suggest that DPP-4 inhibition, in addition to improving metabolic control, have the potential to interfere with the onset and progression of diabetic microangiopathy [153].
5) Pancreatic β-cell dysfunction
  In streptozotocin-induced diabetic rats, a novel long-acting DPP-4 inhibitor, PKF-275-055 at 3, and 10mg/kg significantly reduced glucose excursion during the oral glucose tolerance test, with increases in plasma insulin and active GLP-1 levels as well as decrease in plasma DPP-4 activity [154]. Furthermore, PKF-275-055 significantly inhibited glycated hemoglobin, insulin resistance, gastric emptying and small intestinal transit rates, which were associated with pancreatic β-cell regeneration and decreased apoptosis [154]. In addition, GLP-1 protected beta cell against AGEs-induced apoptosis and necrosis [155]. GLP-1 restored the redox balance, improved the responsiveness to glucose, and attenuated the AGEs-induced RAGE expression pancreatic islet cell line HIT-T 15 [155]. Moreover, GLP-1 restored Nrf2 levels in HIT-T 15 cells and subsequently decreased the susceptibility of β-cells to oxidative stress, which could lead to improvement of insulin synthesis in association with increased expression of MafA and PDX-1, two transcriptional factors that activates insulin gene promoter [156]. Taken together, these findings provide evidence that long-acting DPP-4 inhibitors and/or GLP-1 could protect pancreatic β-cells from the deleterious effects of AGEs.

## Conclusions

As mentioned above, there is a crosstalk between the AGEs-RAGE axis and DPP-4-incretin system in the pathogenesis of diabetes-associated disorders [58,157]. Further longitudinal study is needed to clarify whether DPP-4 inhibitors and/or GLP-1-based therapies could prevent the development and progression of devastating complications of diabetes.
